# The pelvic floor during pregnancy and delivery: Can pelvic floor trauma and disorders be prevented?

**DOI:** 10.1111/aogs.14875

**Published:** 2024-05-19

**Authors:** Torbjørn Moe Eggebø, Ingrid Volløyhaug

**Affiliations:** ^1^ Department of Clinical and Molecular Medicine Norwegian University of Science and Technology Trondheim Norway; ^2^ Department of Obstetrics and Gynecology Stavanger University Hospital Stavanger Norway; ^3^ Department of Obstetrics and Gynecology Trondheim University Hospital Trondheim Norway

The pelvic floor consists of the levator ani muscle, nerves, and connective tissue. The levator ani muscle attaches to the bony pelvis and provides support of the urinary bladder, uterus, vagina and rectum. A healthy and well‐functioning pelvic floor is important in stabilizing the pelvic organs and preventing pelvic floor disorders. The superficial muscles of the perineum and the anal sphincters also contribute to this interaction between muscles, nerves and connective tissue, all subject to injury during childbirth.

“Pelvic floor disorders” is the common term for urinary incontinence, anal incontinence, pelvic organ prolapse, pelvic floor pain, and sexual dysfunction. At least 25% of all women have some of these symptoms and suffer from prolapse and incontinence.^1^ Some women suffer from more than one impairment. Pelvic floor disorders may have a great impact on a woman's self‐image and her social life. These impairments are also an economic burden for society because of sick‐leaves, hospital visits and assessments and conservative and surgical treatment.^1^ The lifetime risk of requiring urinary incontinence or prolapse surgery is approximately 20% in Western countries.^1^ Pregnancy, and in particular vaginal delivery, where pelvic floor, perineal, and anal sphincter trauma may occur, are the main risk factors for pelvic floor disorders later in life.^2^

Pelvic floor muscle training (PFMT) is used as first‐line treatment for women with established urinary incontinence and mild pelvic organ prolapse. It is also often used for women with muscular pelvic pain and sexual dysfunction. Teaching and learning PFMT may be challenging since these muscles are inside the body and muscle movements are barely visible on the perineum. Women with injured pelvic floor muscle have, on average, a weaker contraction than women with intact muscles and nerves, but most women with injuries are still able to contract. Teaching and learning PFMT during pregnancy, when muscles, nerves and connective tissue are intact, is probably easier than starting with a weakened pelvic floor in the postpartum period or later in life when symptoms have occurred. One study suggested that pelvic floor training may be associated with shorter active pushing phase during childbirth,^3^ but few studies have focused on whether PFMT during pregnancy can prevent levator trauma.

In this issue of AOGS, Zhang et al. included 30 studies in a systematic review and investigated the effect of PFMT on the prevention of urinary incontinence and perineal tears.^4^ They included only randomized controlled trials, providing a high level of evidence. The meta‐analysis of 12 studies regarding the prevention of urinary incontinence showed a significant and clinically relevant risk reduction (RR = 0.72) for urinary incontinence for women in the PFMT group. Pelvic floor muscle training was also effective in prevention of third‐ or fourth‐degree perineal tears involving the anal sphincters at childbirth, with a RR of 0.50. These meta‐analyses indicate that PFMT during pregnancy may improve quality of life for many women. Few data exist on the prevention of anal incontinence and this should therefore be included in future trials.^5^ Since PFMT reduces the risk of anal sphincter injury, which is a strong risk factor for anal incontinence, we might assume a protective effect of PFMT, but this still needs to be confirmed.

The dimensions of the levator hiatus increase throughout pregnancy.^6^ Also during the first stage of labor there is a modest stretching of the levator muscle and increased levator hiatal dimensions,^7^ but most stretching and muscle injuries probably occur during the expulsive phase of labor when the fetal head passes through the levator hiatus.^8^ The levator ani muscle has a huge ability to stretch without rupturing, and the medial fibers can stretch 2.5 times their original length.^9^ Still, levator avulsions can occur, and avulsion diagnosed on ultrasound or MRI is defined as an injury to the inferomedial aspect of the puborectalis part of the levator ani muscle complex, when the muscle fibers are detached from their insertion on the symphysis. Such injuries are strongly associated with development of pelvic organ prolapse later in life, and a meta‐analysis confirmed that women with levator avulsions had a two‐fold increased risk of symptomatic prolapse, and four times increased risk of prolapse at clinical examination.^10^ Another meta‐analysis showed that forceps was associated with seven‐fold increased risk of levator avulsion compared to spontaneous vaginal birth and a four‐to five‐fold increased risk compared to vacuum‐assisted birth.^11^ Vacuum carries a similar risk as spontaneous vaginal birth, and such trauma is not seen in women after cesarean section.^11^ These studies provide a strong indication of choice of instrument at operative vaginal delivery, especially to prevent pelvic organ prolapse later in life.

Pudendal nerve injury may cause impairment of the pelvic floor, and multiparity, forceps, increased duration of the second stage, obstetric anal sphincter trauma and high birthweight are important risk factors for nerve injuries.^12^ Pudendal nerve injury usually causes few symptoms because of reinnervation, but in some women, the denervation is more severe and associated with urinary or fecal incontinence.

Instrumental deliveries, and in particular forceps deliveries, in nulliparous women are associated with an increased risk of anal sphincter injuries.^13^ Fetal position and station should be identified precisely before instrumental vaginal deliveries and change of instrument (eg vacuum converted to forceps) should be avoided. Instrumental delivery in the occiput posterior position has the highest risk of anal sphincter injuries, and knowledge about correct traction direction is important.^14^ The Finnish concept of perineal protection (one hand supporting the perineum, the other hand controlling the speed of expulsion, close observation of perineal stretching and communication with the mother) is effective in preventing third and fourth‐ degree perineal tears involving the anal sphincters.^15^ In general, there has been less focus on perineal tears that do not involve the anal sphincters, but some studies have suggested that deeper second‐degree perineal tears are associated with an increased likelihood of perineal pain and lower sexual function compared to women with intact perineum or superficial perineal injuries.^16^ A more detailed classification of second‐degree tears into 2A, 2B and 2C would seem useful in future studies on this topic.^17^

Clinical examinations of fetal station and position during childbirth are subjective and imprecise. Ultrasound has shown high accuracy for assessment of fetal station and position and is recommended as a diagnostic tool before instrumental deliveries.^18^ Ultrasound can also be used in coaching women how to push effectively, and instrumental deliveries may be avoided.^19^ Whether ultrasound during pregnancy and at childbirth can be used to identify women at risk and to prevent pelvic floor and perineal trauma still needs to be investigated.

Pregnant women want to participate in clinical decisions, and they should be informed about risks and preventive procedures. Women increasingly demand predictability about childbirth, risk of pelvic floor and anal sphincter injuries and subsequent consequences. We can all agree that prevention of injuries is better than treatment of subsequent symptoms. We now know that perineal support is protective and that vacuum should be preferred to forceps. Still, further studies regarding the effect of PFMT in preventing not only pelvic floor disorders, but also levator injuries, anal sphincter injuries and second‐degree perineal trauma are needed. The role of pelvic floor ultrasound during pregnancy and childbirth in prevention of birth trauma should also be further explored.
